# Keto Clarity: A Comprehensive Systematic Review Exploring the Efficacy, Safety, and Mechanisms of Ketogenic Diet in Pediatric Epilepsy

**DOI:** 10.7759/cureus.54863

**Published:** 2024-02-25

**Authors:** Youmna Faheem, Amisha Jaiswal, Kainaat Shergill, Kusalik Boppana, Naiela E Almansouri, Saloni Bakkannavar, Ann Kashmer Yu

**Affiliations:** 1 Pediatrics, California Institute of Behavioral Neurosciences and Psychology, Fairfield, USA; 2 Internal Medicine, California Institute of Behavioral Neurosciences and Psychology, Fairfield, USA; 3 General Surgery, California Institute of Behavioral Neurosciences and Psychology, Fairfield, USA; 4 Internal Medicine and Gastroenterology, California Institute of Behavioral Neurosciences and Psychology, Fairfield, USA; 5 Internal Medicine, University of Tripoli, Tripoli, LBY

**Keywords:** medium-chain triglyceride ketogenic diet (mctkd), modified atkins diet (mad), seizure, epilepsy, low glycemic index treatment (lgit), classic ketogenic diet, drug-resistant epilepsy (dre), ketogenic diet, pediatric neurology, pediatrics

## Abstract

Epilepsy, a widespread neurological disorder characterized by recurrent seizures, affects millions globally, with a significant impact on the pediatric population. Antiepileptic drugs (AEDs) constitute the primary treatment; however, drug-resistant epilepsy (DRE), especially in children, poses a therapeutic challenge. Alternative interventions, such as surgery, vagus nerve stimulation, and the ketogenic diet (KD), have been explored. This systematic review aims to investigate various types of KDs, their distinctions, their effectiveness, and their safety concerning the reduction of seizure frequency, achieving seizure freedom, and the occurrence of adverse events. The study adheres to the Preferred Reporting Items for Systematic Reviews and Meta-Analyses (PRISMA) 2020 guidelines. A comprehensive search was conducted using databases such as PubMed Central (PMC), MedLine, and Science Direct to identify relevant articles. Eligibility criteria and quality assessment tools were applied to evaluate the potential risk of bias and select 11 articles for inclusion in this review. The selected articles encompassed four randomized controlled trials (RCTs), two systematic reviews, and five narrative reviews. The data collected for this review was completed on October 2, 2023. Challenges, such as palatability, cultural factors, and adherence difficulties, were identified. Family or caregiver involvement plays a pivotal role in treatment success. Despite numerous RCTs and reviews, information gaps persist, hindering conclusive outcomes. Evaluating the risk-benefit ratio is crucial, considering potential side effects. The highly individualized nature of KD therapy, influenced by diverse seizure types and syndromes, necessitates a trial-and-error approach monitored by a multidisciplinary team. Long-term safety and efficacy demand continuous real-life patient data review. In summary, while KD presents a promising alternative for DRE, its success relies on meticulous planning, individualized implementation, and ongoing research to address existing challenges and information gaps.

## Introduction and background

Drug-resistant epilepsy (DRE), as defined by the International League Against Epilepsy, refers to the condition where two appropriate and tolerated antiepileptic drug (AED) treatments, whether used individually or in combination, fail to achieve sustained seizure freedom. Managing DRE is often challenging, and pharmacological interventions are not as effective, leading to the exploration of alternative treatments like surgery, vagus nerve stimulation, or dietary therapy [1].

For a century now, the ketogenic diet (KD) has been employed as a therapeutic approach for managing epilepsy. This dietary regimen is characterized by its high-fat content, low-carbohydrate intake, and sufficient level of protein to support growth, effectively replicating the metabolic changes that occur during periods of starvation [1-2].

In the early 20th century, fasting gained attention for managing seizures, as noted by Guelpa and Marie (1911) and Geyelin (1921). Geyelin's study linked improved seizure control during fasting to a ketotic effect similar to the KD [3]. Dr. Russell Wilder proposed the KD to prevent malnutrition, and Dr. Mynie Peterman demonstrated its success in children, with 95% showing improved seizure control and 60% achieving freedom from seizures [4]. Despite initial popularity, the discovery of phenytoin in 1938 led to a decline in KD use, replaced by antiseizure medications (ASMs) due to perceived unpalatability, poor adherence, and a shift in effectiveness perception, reducing dietitian training and overall acceptance [5]. Wilder and Peterman (1925) confirmed the KD's effectiveness in seizure control, especially in children. Widely used in the 1930s, it showed greater success in children due to rapid ketone production and better compliance [3]. Despite early support, a lack of controlled trials led to decreased use. Contemporary studies reaffirm its efficacy for refractory epilepsy, even compared to new medications, but the evidence base is limited [3-5].

Studies on the safety of the KD for refractory childhood epilepsy identified over 40 adverse effects, including common ones like gastrointestinal issues, hyperlipidemia, and lethargy [6]. Severe adverse effects were rare, and no deaths were directly linked to the KD. While the KD is generally considered safe, cautious medical supervision and ongoing follow-up are recommended to evaluate its long-term impact on children's overall health [6]. Assessing the efficacy of the KD among pediatric epileptic patients found that those who achieved over 50% seizure reduction were more likely to remain on the diet. This highlights the importance of long-term follow-up, dropout analysis, and improved seizure type characterization in future observational studies [6-9,10].

The resurgence of interest in the KD for the management of refractory childhood epilepsy over the past 15 years has prompted numerous studies to explore its safety, tolerability, and efficacy. This systematic review aims to assess the available evidence from prospective studies on the safety and tolerability of KD as a treatment option for children with refractory epilepsy.

## Review

Methods

The Preferred Reporting Items for Systematic Reviews and Meta-Analyses (PRISMA) 2020 guidelines were followed in this systematic review [11].

Eligibility criteria

Inclusion Criteria

The systematic review includes pediatric-focused randomized controlled trials (RCTs), prospective and retrospective studies, clinical trials, and observational studies published in English and the years 2000-2023. Targeted participants are aged from birth to 18 years old with various epilepsy syndromes, using the KD or its variants. Relevant outcomes include changes in seizure frequency, cognitive improvements, seizure freedom, and diet-related adverse events.

Exclusion Criteria

Excluded are case reports, case series, studies lacking primary data, and those involving adults or animals. Studies exploring dietary interventions other than the KD are not considered. Additionally, studies published before 2000 or with unspecified publication dates, as well as those in languages other than English without translations, are excluded.

Search strategy and databases

The databases PubMed Central (PMC), MedLine, and Science Direct were explored on October 2, 2023, for potential articles published from the years 2000 to 2023. Using the Boolean method, Medical Subject Heading (MeSH) terms were combined with keywords to identify all potentially relevant articles pertaining to the efficacy and safety of KD in the pediatric population. The data collected for this review was completed on October 2, 2023.

The keywords used to conduct the search were epilepsy, ketogenic diet (KD), childhood epilepsy, drug-resistant epilepsy (DRE), classic ketogenic diet (cKD), medium-chain triglyceride (MCT), low glycemic index (LGI), modified Atkins diet (MAD), and refractory epilepsy. The EndNote reference manager was used to group all the references and remove duplicates. Irrelevant studies were then excluded based on titles and abstracts. Following this, full-text papers were reviewed for further exclusion. The details of the search strategy are listed in Table 1 below.

**Table 1:** The strategy of the database search with their respective filters and results

Database	Search strategy	Filters applied	Results
PubMed Central	("Epilepsy"[Mesh]) AND "Epilepsy/diet therapy"[Mesh]) AND ("Epilepsy/diagnosis"[Mesh] OR)) AND ("Epilepsy/complications"[Mesh] OR)) AND ("Epilepsy/therapy"[Mesh])	Free full text, human, English, child: birth-18 years	277
MedLine	Keywords: "Epilepsy" AND "Ketogenic Diet"	2000-2023	209
Science Direct	Keywords: childhood epilepsy; drug-resistant epilepsy; ketogenic diet; low glycemic index; modified Atkins diet; refractory epilepsy	2000-2023, open access, and open archive	450

Data Collection Process and Data Items

In our study, we employed a method of data extraction from research reports that involved using standardized piloted forms. Two independent reviewers were assigned to each report, and they extracted data separately. After data extraction, a third reviewer was designated to resolve any discrepancies or disagreements between the two initial reviewers. Additionally, to ensure data accuracy, we contacted the primary investigators of the included studies for clarification on specific data points and to confirm the accuracy of the information extracted from their reports. This process was conducted for each included study to enhance the reliability of our data collection.

Risk of bias in individual studies

Assessment of Risk Bias

We evaluated the risk of bias in individual studies using established criteria. We used the Cochrane Collaboration risk of bias tool (CCRBT) for randomized controlled trials [12], the scale for the assessment of narrative review articles 2 (SANRA 2) for narrative reviews [13], and the assessment of multiple systematic reviews 2 (AMSTAR 2) for systematic reviews [14].

This assessment was performed at the study level, and two independent reviewers conducted the evaluations. Disagreements between the two reviewers were resolved through discussion or consultation with a third reviewer.

Utilization of Risk of Bias Information

The risk of bias assessments for individual studies will be considered in the data synthesis and interpretation of our findings. Studies with a high risk of bias in critical domains will be excluded from the study. We will conduct sensitivity analyses to assess the robustness of our results. This approach allows us to account for the quality and reliability of the included studies when drawing conclusions from the synthesized data.

Quality assessment was done for the full-text articles using tools specific to each type of study. Studies with a score of >70% were finalized to be used in the paper. Details of the quality assessment and the tools used for the final articles accepted for this review are listed in Table 2 as seen below.

**Table 2:** Details of the quality assessment and the tools used for the final articles accepted for this review CCRBT: Cochrane Collaboration risk of bias tool; RCTs: randomized controlled trials; SANRA 2: scale for the assessment of narrative review articles 2; AMSTAR 2: assessment of multiple systematic reviews 2

Quality assessment tool	Type of study	Total score	Accepted score (>70%)	Number of accepted studies (#)
CCRBT	RCTs, clinical trial	7	5	4: Sondhi et al. [15], Neal et al. [16], Kim et al. [17], and Sharma et al. [18]
SANRA 2	Narrative review	12	9	5: Zarnowska [4], Wells et al. [5], Newmaster et al. [19], Ułamek-Kozioł et al. [20], and Thiele [21]
AMSTAR 2	Systematic review	16	12	2: Lima et al. [22] and Cross [23]

Results

*Study Selection and Quality Assessment* 

Using three databases (PMC, MedLine, and Science Direct), a total of 936 results were obtained. These results were grouped to remove duplicates, which were 288 in number. The remaining 648 records were screened by title and abstract, and 608 irrelevant records were excluded. There were 40 reports left, which were thoroughly screened as full-text papers. Out of those, 28 were excluded. Quality assessment was then performed on the remaining studies, using tools specific to each type of study. This was done independently by two authors. Eleven final studies with a score of >70% were included in this review. The studies constituted four RCTs, two systematic reviews, and five narrative reviews.

**Figure 1:**
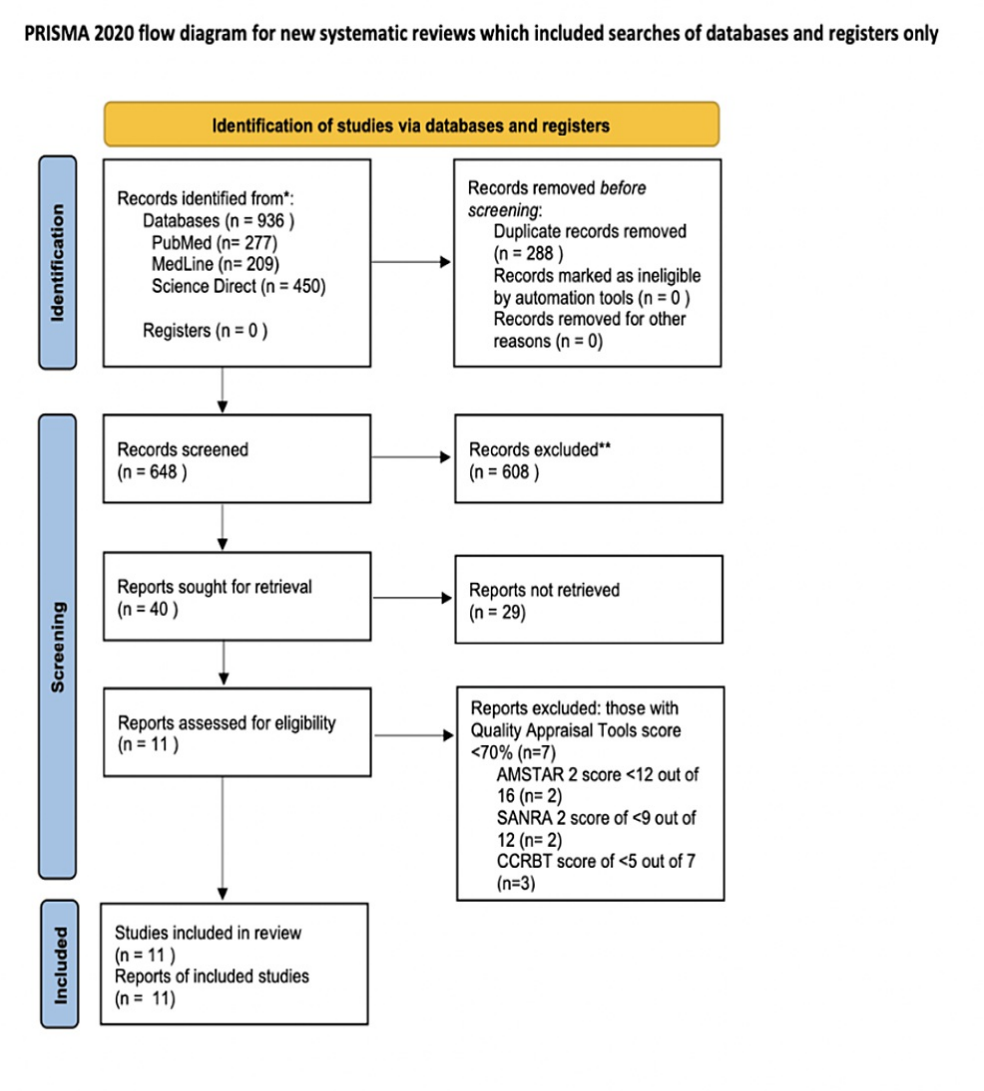
PRISMA flowchart PRISMA: Preferred Reporting Items for Systematic Reviews and Meta-Analyses; AMSTAR 2: assessment of multiple systematic reviews 2; SANRA 2: scale for the assessment of narrative review articles 2; CCRBT: Cochrane Collaboration risk of bias tool

The last date for data collection was October 2, 2023. A PRISMA flow diagram representing the search selection is shown in Figure 1 below.

The main features of the RCTs chosen for this review are listed in Table 3 below.

**Table 3:** Main features of the RCTs chosen for this review. A total of four RCTs were included in this review, the details of which are summarized in this table RCTs: randomized controlled trials; KD: ketogenic diet; MCT: medium-chain triglyceride; MAD: modified Atkins diet; MCKTD: medium-chain triglyceride ketogenic diet; LGIT: low glycemic index treatment; cKD: classic ketogenic diet

Author and year	Study type	Inclusion criteria	Sample size (dropouts)	Intervention	Outcomes
Sondhi et al. 2020 [15]	RCT	The study included 170 children (1-15 years old) meeting the criteria: experiencing ≥4 seizures monthly, resistance to ≥2 antiseizure medications, and no prior exposure to KD, MAD, or LGIT	170 (12)	Study participants were randomly assigned to either KD, MAD, or LGIT while continuing their antiseizure drug therapy	LGIT diet showed better seizure reduction and fewer adverse events compared to KD and MAD, emphasizing personalized risk-benefit decisions for dietary interventions
Neal et al. 2008 [16]	RCT	Ages ranging from two to 16 years, experiencing a minimum of seven seizures weekly, showing resistance to at least two anticonvulsant medications, and having no prior exposure to the KD	145 (89)	Children with uncontrolled epilepsy were assigned classical or MCT diets, with seizure frequency, treatment discontinuation, diet tolerance, and blood ketone levels monitored at three, six, and 12 months	No significant differences in seizure reduction percentages were observed at three, six, and 12 months between the cKD and MCTKD, with comparable responder rates, side effects, and reasons for discontinuation for both approaches
Kim et al. 2016 [17]	RCT	Patients aged between one and 18 years, experiencing a seizure frequency of more than four seizures per month, and showing resistance to at least two prescribed antiepileptic drugs	104 (34)	The study assessed the effectiveness, safety, and tolerance of MAD and cKD in treating refractory childhood epilepsy through random allocation of pediatric patients with treatment-resistant epilepsy to either dietary group	The study recommends the MAD as the primary treatment for intractable epilepsy in children, with the cKD potentially preferred for patients under two years old requiring rapid brain stabilization due to its higher fat ratio, while also highlighting MAD's comparable effectiveness and better tolerability
Sharma et al. 2013 [18]	RCT	The study investigated refractory epilepsy cases in children aged six months to five years, with at least daily or seven seizures per week, despite trying at least three antiepileptic drugs, excluding those with metabolic disorders or surgically treatable epilepsy sources	41 (31)	The study utilizes a KD to treat refractory epilepsy in young Indian children, including pre-diet preparation, gradual introduction, and assessments to measure its impact on reducing seizures and improving patient well-being	The KD controlled seizures in over half of children for six months but led to discontinuations due to concerns like decreased serum albumin and increased urinary calcium-to-creatinine ratio, underscoring the need for ongoing medical supervision

The main characteristics of the narrative and systematic reviews are listed in Table 4 below.

**Table 4:** The main characteristics of the narrative and systematic reviews. A total of five narrative reviews and two systematic reviews were included in this review, the details of which are summarized in this table NR: not reported; KD: ketogenic diet; cKD: classic ketogenic diet; MAD: modified Atkins diet; MCTKD: medium-chain triglyceride ketogenic diet; LGIT: low glycemic index treatment

Author and year	Study type	Inclusion criteria	Key points
Zarnowska 2020 [4]	Narrative review	NR	The KD is highly effective for controlling seizures in challenging epilepsy cases, with sustained benefits across ages and syndromes, attributed to its tolerability and manageable short-term side effects, particularly in the initial treatment phase
Wells et al. 2020 [5]		Studies investigating the impact of KD therapy on refractory epilepsy outcomes in children and adolescents. Excluded conference papers, reports, and case studies	The cKD offers substantial seizure reduction and potential long-term freedom, the MAD achieves over 50% reduction, especially in young children, and the MCTKD is effective even in super-refractory cases, though long-term efficacy data for the LGIT are limited
Newmaster et al. 2022 [19]		NR	Monitoring for complications related to the KD requires consideration of risk factors, and temporary issues are common during long-term treatment, emphasizing the need for multidisciplinary oversight and fat-to-carbohydrate ratio adjustments under pediatric neurologist guidance
Ułamek-Kozioł et al. 2016 [20]		Children and adolescents, diagnosed with medication-resistant epilepsy, who have not attained satisfactory seizure management despite the use of at least two antiepileptic medications	Around 15% of KD patients become seizure-free, with approximately 33% experiencing a 50% or greater reduction in seizures, while recent research highlights concerns regarding its potential negative effects on lipoprotein subfractions in epilepsy patients
Thiele 2003 [21]		A comprehensive review of literature spanning the last 80 years was conducted, specifically examining data related to the KD. This review emphasized aspects such as its effectiveness, how well it is tolerated, the age groups of patients involved, and the methodologies employed in the studies	Many prospective and retrospective studies have shown the KD's remarkable efficacy in treating epilepsy, particularly in children. However, the absence of class 1 studies limits a more precise understanding of the diet's role in epilepsy management
Lima et al. 2022 [22]	Systematic review	The systematic review explored the impact of KDs on cognitive function in individuals with pharmacoresistant epilepsy, encompassing various study types and diet protocols with MCT supplementation, without age or gender restrictions	The majority of studies (19 out of 24) indicated improved cognitive function associated with KD treatment across various cognitive domains, but challenges in drawing definitive conclusions remain due to diverse assessment methods and methodological limitations
Cross 2015 [23]		Out of 259 studies, 45 articles, including six systematic reviews and seven RCTs, were reviewed, meeting criteria such as English publications, open-label or single-blinded designs, at least 20 participants per group, over 80% follow-up, and reporting outcomes after a minimum three-month follow-up from treatment initiation, with longer-term outcomes considered if available	Behavioral and psychological treatments improve quality of life and alleviate anxiety and depression in epilepsy, while KDs are evaluated based on seizure frequency, quality of life, and adverse effects

Discussion

This section will discuss the different types of KDs and their characteristics, the efficacy of each type of KD, different theories on its mechanism of action, adverse effects, safety, and complications.

In assessing dietary therapies for childhood DRE, including cKD, MAD, medium-chain triglyceride ketogenic diet (MCTKD), and low glycemic index treatment (LGIT), all showed short-term effectiveness. The MAD stood out with superior tolerability, a higher likelihood of a 50% or greater reduction in seizures, and comparable chances of a 90% or greater reduction, making it a promising alternative to the KD. However, further direct comparative studies are needed to validate these findings and determine the optimal dietary approach for managing DRE in children [5,7,8,15].

Types of KD

cKD

This dietary approach involves a high-fat ratio, with a fat-to-combined protein and carbohydrate ratio of 3-4:1, contributing to 75-80% of daily calories from fat. Initiated in a hospital, it is prescribed by dietitians or neurologists, following a dietary ratio-based methodology. Fluid intake is determined based on body weight, and meticulous food weighing is required. Additionally, laboratory evaluations are essential, and supplementation of vitamins and minerals is prescribed. The approach emphasizes low glycemic control as part of the therapeutic strategy [24-26].

MCTKD 

This dietary approach involves a variable high-fat ratio, utilizing medium-chain triglycerides (MCT) for ketone production. There is typically no fluid restriction, and initiation occurs in a hospital setting, prescribed by dietitians or neurologists. The percentage of MCT in the diet is adjusted, and meticulous food weighing is required, along with essential laboratory evaluations. Additionally, vitamin and mineral supplements are prescribed, and the approach emphasizes low glycemic control [24-26].

MAD

This diet is high-fat, low-carbohydrate with no specific ratio or food weighing. It involves carbohydrate restriction (starting at 10 g/d for children) and typically has no fluid restriction. Initiation can occur in an outpatient clinic, and there are no specific meal plans. The approach allows dietary flexibility for protein and fat, with potential vitamin and mineral supplementation. It emphasizes low glycemic therapy [24-26].

LGIT

This diet limits carbohydrate intake to 40-60 g/d, maintaining a balanced macronutrient profile with higher fat (60%) and protein (20-30%) intake. All carbohydrates chosen have an LGI, and there is no fluid restriction. A dietitian's prescription is required, and while there are no specific meal plans, recommendations are provided. Food portion sizes are determined by diabetic exchanges, and supplementation with vitamins and minerals is based on individual needs. The approach focuses on low glycemic therapy to stabilize glucose levels [24-26].

Table 5 shows the macronutrient distribution across different KD types.

**Table 5:** Macronutrient distributions across KD types KD: ketogenic diet; KDT: ketogenic diet therapy; MAD: modified Atkins diet; MCTKD: medium-chain triglyceride ketogenic diet; LGIT: low glycemic index treatment; MCT: medium-chain triglyceride; LCT: long-chain triglyceride References: [4,5]

KDT	Clinical implementation	Diet pattern	Percent of the total daily energy intake		
			Fat (%)	Carbohydrate (%)	Protein (%)
KD	Inpatient stay	KD ratio of 3:1-4:1	90	4	6
Traditional MCTKD	Inpatient stay	60% of the total energy intake from MCT	70-75	15-18	10
Modified MCTKD	Inpatient stay	30% of the total energy intake from MCT and 30% from LCT	70-75	15-18	10
MAD	Outpatient	KD ratio of 1:1-2:1	60-65	5-10	30
LGIT	Outpatient	40-60 g carbohydrate per day	60	10	30

The mechanisms of the KD

The KD restricts carbohydrate intake to less than 60 grams per day, promoting ketosis by reducing blood glucose and insulin release. Ketosis utilizes gluconeogenesis in the liver and free fatty acids (FFAs) as the primary energy source when the insulin-to-glucagon ratio drops. The brain uses ketone bodies from FFAs when glucose is scarce, supplying up to 60% of its energy. Nutritional ketosis maintains stable blood glucose, normal blood gas values, and elevated ketone bodies and lipids, differentiating it from diabetic ketoacidosis. It's considered an adaptive mechanism supporting brain and muscle energy needs, possibly significant in human evolution.

While measures of ketosis may not consistently correlate with seizure control, they indicate adherence to the diet. The exact reasons for the KD's neurological benefits remain unclear, with theories suggesting efficient energy for brain development and repair. Factors like FFAs, glucose levels, and their relation to seizure control require further study for tailored treatments [27,28].

Biochemical Effects of the KD

The KD mimics fasting, shifting the body's energy production to ketones. Beta-hydroxybutyrate (BHB) is a key marker of KD adherence. However, BHB levels do not significantly correlate with seizure activity, indicating that ketosis might not directly influence seizure frequency [4,5].

Impact on Neurotransmitters

Ketosis may protect against seizures by modulating glutamate and gamma-aminobutyric acid (GABA) neurotransmitters. The KD potentially enhances glutamate recycling, increases GABA synthesis, reduces reactive oxygen species (ROS), and promotes energy generation. Higher GABA levels can inhibit neuronal excitation through chloride channel receptors, leading to hyperpolarization [4,5].

Mitochondrial Function and ROS Production

The KD affects mitochondrial function by lowering ROS production. Animal studies show increased mitochondria, reduced ROS, and elevated uncoupling proteins in the hippocampus with a KD. Ketone bodies decrease ROS and enhance glutathione levels, an intracellular antioxidant protecting neurons and inhibiting cell death [4,5].

Anti-inflammatory Effects

Inflammation and brain damage are interconnected with the development of epilepsy. Ketosis can aid in reducing neuroinflammation and protecting neurons from cell death. BHB has been shown to inhibit the production of nucleotide-binding domain, leucine-rich-containing family, pyrin domain-containing-3 (NLRP3) inflammasomes, which are involved in inflammatory processes. BHB's interaction with the hydroxycarboxylic acid 2 (HCA2) receptor also contributes to neuroprotection and anti-inflammatory effects [4,5].

Gut-Brain Axis

The gut-brain axis, crucial for communication between the central and enteric nervous systems, is influenced by ketosis through gut microbiota modification. The KD alters the microbial structure, producing short-chain fatty acids (SCFAs) like butyrate and acetate, impacting the gut-brain axis. Gut microbiota plays a significant role in neurological disorders, including epilepsy, with KD treatment showing promise. Studies suggest gut microbiota may mediate KD's protective effects against seizures. Changes in microbiota composition, like increased *Parabacteroides* and *Akkermansia muciniphila*, are linked to seizure protection. KD treatment in infants with DRE rapidly modifies gut microbiota, reducing seizure frequency. Research on KD's impact on the gut microbiota in conditions like GLUT1 deficiency syndrome reveals changes in composition. Studies explore the use of prebiotics or probiotics in KD therapy to restore a healthy balance in gut dysbiosis. KD treatment in children with DRE affects both taxonomic and functional profiles of gut microbiota, emphasizing the need for further research on specific alterations essential for therapeutic effects. The KD, influencing neuronal excitability through mechanisms like gut microbiota changes, prompts the exploration of dietary supplements such as probiotics and prebiotics. Continued research is crucial for a comprehensive understanding of the mechanisms behind KD treatment for epilepsy and neurological disorders [4,5,29,30].

Adipokine Alterations in Children with DRE Undergoing KD Treatment

A study delved into the serum concentrations of adipokines, bioactive molecules secreted by adipose tissue with roles in metabolic regulation and inflammation. Specifically, the focus was on three key adipokines, adiponectin, omentin-1, and vaspin, renowned for their metabolic benefits and anti-inflammatory properties. The investigation aimed to ascertain how these adipokines were affected in children with DRE undergoing KD treatment. To provide a comprehensive evaluation, the KD group's results were compared with those of children receiving valproates (VPA), a common epilepsy medication, and a control group of children with headaches. Additionally, the researchers explored potential correlations between adipokine concentrations and various anthropometric and metabolic parameters.

The findings revealed significant alterations in the levels of these specific adipokines when comparing children on the KD with those in the VPA group and the control group. This suggests that the KD not only impacts the body's metabolism but also influences inflammation through changes in adipokine concentrations. These modifications in adipokines may play a pivotal role in the therapeutic effects of the KD, known for its effectiveness in reducing epileptic seizures. This newfound insight into the interaction between the KD, adipokines, and epilepsy paves the way for further exploration of the diet's mechanisms and its potential for innovative therapeutic approaches in managing DRE in children.

In the study, the KD group exhibited significantly higher levels of adiponectin and omentin-1 compared to both the VPA group and the control group, while vaspin concentrations were significantly lower in the KD group compared to the other groups. However, correlation analysis did not identify significant relationships between serum adipokine concentrations and the number of seizures, anthropometric measurements, or biochemical parameters in children treated with the KD [31].

Efficacy

The KD has exhibited its efficacy as a treatment for epilepsy across diverse age groups, spanning from infancy to adulthood. Notably, it has proven particularly effective in children under the age of two, playing a crucial role in preventing irreversible brain damage in this vulnerable group [32-34]. The KD is primarily deployed when dealing with refractory epilepsy cases where two ASMs have proven futile. Several conditions display a notable positive response to the KD, with response rates surpassing 70%. These conditions include Dravet syndrome, infantile spasm, and tuberous sclerosis complex. Additionally, the KD shows moderate benefits in conditions like childhood absence epilepsy, cortical malformations, juvenile myoclonic epilepsy, Rett syndrome, Landau-Kleffner syndrome, and Lennox-Gastaut syndrome (LGS) [34-36]. Remarkably, for specific conditions such as glucose transporter type 1 deficiency syndrome (GLUT1 DS) and pyruvate dehydrogenase complex deficiency (PHDH) syndrome, the KD is considered the primary therapy of first choice.

Beyond the immediate period, the KD continues to provide benefits, as studies have shown significant reductions in seizure frequency three to six years post initiation. These reductions have allowed for a decrease in the number of anticonvulsant drugs taken by many patients. The Cochrane Database analysis further highlights the KD's efficacy, reporting impressive seizure freedom rates after three months, reaching up to 55% for the classical KD 4:1 ratio and up to 85% with a reduction of over 50% of seizures in up to 85% of children. Similarly, the MAD displayed substantial efficacy, with 50% of patients achieving a 50% reduction in seizures and 28% experiencing over a 90% reduction [37].

The KD not only controls seizures but also exhibits neuroprotective and disease-modifying properties, leading to improvements in the quality of life. It extends its positive effects to neurobehavioral development, cognitive functions, and sleep quality. The KD has been shown to decrease sleep and improve sleep quality by increasing rapid eye movement (REM) sleep, significantly impacting the overall well-being of patients [32]. In some instances, patients can reduce the number of ASMs, although some concerns about ASM dosage adjustments exist and the evidence surrounding these adjustments remains mixed [38-40].

The efficacy of different KD therapies is summarized in Table 6 below.

**Table 6:** Efficacy of different KDTs in managing seizures KD: ketogenic diet; KDT: ketogenic diet therapy; cKD: classic ketogenic diet; MCTKD: medium-chain triglyceride ketogenic diet; MAD: modified Atkins diet; LGIT: low glycemic index treatment

KDT	Seizure reduction at different time points	Seizure freedom	Comments
cKD	52% at three months, 43% at six months, 40% at 12 months, 33% at 24 months, and 30% at three years [41]	55% freedom and 85% seizure reduction on a 4:1 diet at >3 months where at least 50% of children have at least a 50% reduction in seizures at six months [30]. 15 (85%) had greater than 50% reduction in seizures at >3 years [39]	Promising long-term efficacy
	A study with a high number of patients with Lennox-Gastaut syndrome had better seizure control on a 4:1 rather than a 3:1 KD ratio [42,43]		
	58% at three months (4:1) and 63% at three months (2.5:1) [44]		A 2.5:1 KD ratio may be as effective as 4:1 KD
cKD in infants	68% at one month, 82% at six months, and 91% at 12 months	With 20% at one month, 29% at six months, and 27% at 12 months achieving seizure freedom	It is recommended to start on a 1:1 KD diet and then adjust based on ketosis and tolerance [36]. The cKD with a 3:1 ratio is used in infants to meet protein requirements; children younger than two years of age may be an ideal age population to start the cKD [31]. Safe and effective for infants as young as six weeks [45]
Modified MCTKD	No significant difference in seizure reduction compared to cKD; 64.3% reported >50% seizure reduction by month 3	27.6% became seizure-free	Some studies report positive results [46,47]
MAD	>30% with >50% reduction in seizures in several studies [18,31,48]		Families found the diet very restrictive and had difficulty finding recipes suitable for the children. Overall efficacy was similar to cKD, except for 1-2-year-olds, but the results were statistically insignificant. MAD had a larger decrease in seizure frequency than cKD for those >2 years of age, but the opposite in those <2 years of age. Quality of life may improve in responders (increased from 43% to 68%) but decreased from 49% to 39% in non-responders
LGIT	Seizure reductions of greater than 50% from baseline at one, three, six, nine, and 12 months measured 42%, 50%, 54%, 64%, and 66%, respectively, at the follow-up intervals [48-50]		Efficacy did not differ significantly with regard to seizure type. Promising but limited long-term data

KD and Growth

In a study including 45 children (23 males, 22 females) with refractory epilepsy, aged eight months to 17.3 years, the primary regimen was the classical KD 4:1 ratio, with some receiving variations 2:1 or 3:1. Administration methods varied, with most fed solid food and a minority through a tube. Growth assessment showed normal weight and height patterns for the majority, with a limited subset experiencing growth retardation, defined by a deviation of -1 standard deviation score (SDS) or more, during the first and second years on the KD [51].

Conflicting Reports

There are varying reports on the impact of the KD on the linear growth of children. Some studies have suggested a decrease in height percentiles or height Z-scores in a significant portion of children after a certain period on the diet. For example, Williams et al. and Peterson et al. reported a decline in height percentiles and Z-scores, with Neal et al. noting a more pronounced drop in height among boys [52,53].

Mixed Findings

On the other hand, some studies, including Kim et al. and Vining et al., found no significant changes or even improvements in BMI Z-scores. Vining et al. observed a decline in height Z-scores after six months on the KD, particularly in younger children, while this study did not find significant differences when considering age [54,55].

Growth Deceleration

Spulber et al. found that during the first year on the KD, growth velocity decreased significantly in children with refractory epilepsy, causing a drop in height SDS. This decrease in height SDS correlated negatively with blood BHB levels and positively with serum IGF-1 levels [56].

Growth Monitoring and Mechanism of Growth Delay

The study suggests careful growth monitoring for children with refractory epilepsy on KD treatment, using a cut-off point of -1 SDS to assess linear growth. Adjustments to the diet, including calorie-to-protein ratio, micronutrients, and the ketogenic ratio, can be guided by this evaluation. The exact mechanism of growth delay during KD treatment is unclear, with proposed factors including caloric restriction, metabolic acidosis (β-OHB levels), and low IGF-1 levels. Some studies indicate that more significant ketosis may be associated with delayed growth, while moderate ketosis may have a less pronounced impact. 

Ferraris et al. conducted a retrospective study to assess the impact of the KD on the linear growth of 34 patients. The study found that 20% of the children in the study exhibited growth retardation at 12 months after being on the KD. In a study, which is not explicitly mentioned by the author's name, using a criterion of -1 SDS as a cut-off point, growth deceleration was observed in 9% of the patients, which amounted to four individuals. These results highlight the variability in the impact of the KD on linear growth among individuals with epilepsy, and the extent of growth deceleration may vary between different studies and patient populations. Careful monitoring and assessment of individual responses to the KD are crucial to manage and address potential growth-related concerns in children with epilepsy on this treatment [51,56-60]. The adverse effects, complications, short- and long-term effects, as well as safety concerns are summarized in Table 7 below.

**Table 7:** Adverse effects of long-term KDT GERD: gastroesophageal reflux disease; KD: ketogenic diet; cKD: classic ketogenic diet; KDT: ketogenic diet treatment; MCTKD: medium-chain triglyceride ketogenic diet; CVD: cardiovascular disease; DEXA: dual X-ray absorptiometry References: [5,15,16,20,37,50,56,61-63]

System	Adverse effects	Complications	Short-term effects	Long-term effects	Safety concerns
Gastrointestinal	Constipation. Gastrointestinal disturbances. Exacerbation of GERD. Vomiting. Nausea	Constipation, gallstones, and hepatitis	Occurs in 45–48% of patients on 4:1 cKD. Higher incidence with stricter KDTs. Vomiting common in MCTKD. Reported in 25% of Thai children on MCTKD	Constipation may persist. Gastrointestinal issues. May require medication. Dietary changes may help	Addressed by providing an enema, polyethylene glycol, or modifying dietary fibers. Reduce or stop fiber intake to alleviate symptoms. Adjust the KD ratio or provide intravenous fluids. Monitor for gastrointestinal symptoms
Cardiovascular	Elevated serum lipids. QT interval prolongation	High triglycerides and cholesterol. Alternations in arterial function. Cardiomyopathy. Potential cardiac abnormalities	Triglycerides and cholesterol slightly higher on KDs. No significant change in QT interval	Unclear long-term effects. Possible metabolic derangements	Monitor lipid profiles and CVD risk factors. Ensure adequate magnesium and selenium intake
Renal/genitourinary	Renal calculi. Acidosis. Dehydration. Excessive ketosis. Hyperuricemia. Ketosis-induced nephrolithiasis	Uric acid or calcium stones	Occurs in some children on cKD. Need for potassium citrate supplementation	Incidence may increase with long-term use. Monitoring is recommended	Prescribe potassium citrate to prevent stones. Monitor for kidney stones and provide intervention
Skeletal	Decreased bone mineral density. Bone health declines with age	Potential for bone health decline. Decreased bone mineral density. Bone fractures. Osteopenia or osteoporosis risk	Mixed results; some studies show bone mineral loss. Varied findings on bone health	Impacts on bone composition and mineral content. Vitamin D deficiency. Enzyme deficiencies. The need for interventions to prevent bone loss	Regular DEXA scans for bone health. Monitor bone health and consider supplements
Growth	Impaired growth in some cases. Weight loss. Varying impact on growth	Height percentiles may decline. The growth rate may decrease in some cases	Negative impact in calorie-restricted cKD. More liberal diets may have less impact	The effect varies with dietary restrictions. Growth may be different from normal patterns	Individualized dietary plans and supplementation. Monitor growth and nutritional status
Other	Fatigue. Food refusal/decreased appetite. Hypoglycemia. Bruising. Allergy		Hypomagnesemia. Hyponatremia. Hypocalcemia	Secondary carnitine deficiency	

Limitations

This systematic review has limitations: challenges with tolerability, palatability, and participant engagement, especially with children and parents; the lack of blinding due to the nature of dietary interventions and close parental involvement; reliance on caregiver logs for seizure tracking may lead to omissions; a heterogeneous study population introduces potential variability; adapting the KD to local preferences can be challenging; patient adherence is influenced by motivation, perception, palatability, and family cooperation; barriers like limited knowledge, food access, family support, and financial constraints hinder adherence; healthcare system obstacles include lack of support, reimbursement issues, and a shortage of trained personnel; and the keto team may face challenges such as lack of knowledge, disagreements, peer pressure, inadequate communication, and time-consuming activities without reimbursement. Further research is needed to understand KDs in epilepsy management, considering the complexity, challenges, and potential benefits for advancing knowledge and improving epilepsy management.

## Conclusions

The KD shows promising therapeutic benefits for epilepsy and neurological disorders, demonstrating seizure reduction and potential neuroprotective effects. However, careful monitoring for potential adverse effects, especially growth concerns in children, is crucial. A tailored, multidisciplinary approach for each child is essential. Further research is needed to understand the KD's efficacy, safety profile, and impact on growth in children with epilepsy. Individualized care, considering specific conditions and treatment responses, is vital in realizing the potential of the KD in neurological disorders.
